# Fabrication of Novel Ball-Like Polystyrene Films Containing Schiff Base Microspheres as Photostabilizers

**DOI:** 10.3390/polym10111185

**Published:** 2018-10-24

**Authors:** Emad Yousif, Dina S. Ahmed, Gamal A. El-Hiti, Mohammad Hayal Alotaibi, Hassan Hashim, Ayad S. Hameed, Ahmed Ahmed

**Affiliations:** 1 Department of Chemistry, College of Science, Al-Nahrain University, Baghdad 64021, Iraq; 2 Department of Chemistry, College of Science, Tikrit University, Tikrit 34001, Iraq; dinasaadi86@gmail.com (D.S.A.); ch@sc.nahrainuniv.edu.iq (A.S.H.); 3 Department of Optometry, College of Applied Medical Sciences, King Saud University, P.O. Box 10219, Riyadh 11433, Saudi Arabia; 4 National Center for Petrochemicals Technology, King Abdulaziz City for Science and Technology, P.O. Box 6086, Riyadh 11442, Saudi Arabia; mhhalotaibi@kacst.edu.sa; 5 Department of Physics, College of Science, Al-Nahrain University, Baghdad 64021, Iraq; hassan.albattat@gmail.com; 6 Polymer Research Unit, College of Science, Al-Mustansiriyah University, Baghdad 10052, Iraq; drahmed625@gmail.com

**Keywords:** ball-like polystyrene films, carbonyl group index, photo-oxidation, Schiff base, photodegradation, surface roughness

## Abstract

Polystyrene films containing a low concentration of three highly aromatic Schiff bases were prepared using the casting method. The polystyrene films were irradiated with ultraviolet light (300 h). The polystyrene infrared spectra, weight loss, molecular weight reduction and the surface morphology were examined upon irradiation. The Schiff bases acted as photostabilizers and reduced the photodegradation of polystyrene films to a significant level in comparison to the blank film. The images recorded of the surface of the miscible polystyrene/Schiff base blends showed novel ball-like microspheres with a diameter of 3.4–4.3 µm. The Schiff bases were able to endow excellent protection to polystyrene against ultraviolet irradiation.

## 1. Introduction

Polystyrene (PS) has a molecular weight of 100,000–400,000 and is considered to be one of the most commonly used thermoplastic polymers [1]. PS is hard, clear and can be produced as a foam or a solid from polymerization of styrene. It has the advantage of being cheap, lightweight, chemically inert, resistant to acid and base and has the ability to accommodate various deposits [2,3]. However, it is non-biodegradable, flammable and soluble in various chlorinated solvents. It has a range of commercial applications which vary from simple packaging to advanced engineering. [4] The arrangement of the phenyl groups along the polymer chain controls the crystallinity of PS [5]. Atactic PS is very important commercially and has an amorphous structure due to the random arrangement of the phenyl groups along the polymer backbone. However, syndiotactic PS is crystalline and has the phenyl groups distributed alternatively on both sides of the polymeric chain [1]. The chemical and physical properties of PS could be altered if exposed to sunlight in the presence of oxygen for a long period of time. The photo-oxidation of PS produces various functional group fragments which change the solubility, color and mechanical properties, e.g., cracking, brittleness, stiffness and embrittlement [6,7]. It is therefore important to take steps towards improving the chemical behavior of the polymeric materials to refine its suitability for outdoor applications.

The incorporation of additives within the polymeric materials is commercially an important process. The additives used should be colorless, non-volatile, harmless, cheap and compatible with the polymeric materials [8]. Non-biodegradable polymers such as PS can be mixed with a number of additive materials, such as plasticizers, stabilizers and colorants to reduce the rate of photodegradation [9,10]. Such additives act mainly as ultraviolet (UV) stabilizers, radical scavengers, quenchers, antioxidants, flame retardants and antistatic agents [11,12,13,14]. The most common additives used to stabilize PS against photodegradation are heterocycles [15,16,17,18,19,20,21], metal complexes [22,23] and aromatics [24,25,26].

Recently, we showed that Schiff bases could act as an effective poly(vinyl chloride) photostabilizer upon UV irradiation [27]. These Schiff bases acted as UV absorbers and stabilizers and could be added to PS to enhance its resistance to photodegradation. In the current work, we report the efficient use of highly aromatic Schiff bases at low concentration as photostabilizers to reduce photodegradation of PS films when exposed to UV irradiation for a long period (300 h) as part of our research into photostabilization of polymeric materials [28,29,30,31,32,33]. The fabricated PS polymeric materials containing Schiff base microspheres showed novel ball-like polystyrene films.

## 2. Materials and Methods

### 2.1. General

Polystyrene was obtained from the Sigma-Aldrich Chemical Company (Gillingham, UK). The Fourier Transform Infrared (FT-IR) spectra (4000–400 cm^−1^) were recorded on a Jasco FT/IR-4200 spectrometer (Tokyo, Japan). The irradiation of PS films (25 °C, λ_max_ = 365 nm, 6.43 × 10^−9^ ein dm^−3^ s^−1^) was performed using an accelerated weather-meter QUV tester that was purchased from Q-Panel Company (Homestead, FL, USA). Atomic force microscopy (AFM), using a Veeco instrument (Plainview, NY, USA), was used to inspect the surface of PS films. Scanning electron microscopy (SEM) of the PS surface was detected using an Inspect S50 microscope (FEI Company, Czechia, Czech Republic) at an accelerating voltage of 15 KV. A Meiji Techno Microscope (Tokyo, Japan) was used to record the microscopic images of the PS surface. The thickness of PS films (ca. 40 µm) was measured using a Digital Vernier Caliper 2610 A micrometer (Vogel GmbH, Kevelaer, Germany) and the films were fixed using 0.6 mm thick aluminum plate stands (Q-Panel Company, Homestead, FL, USA).

### 2.2. Schiff Bases ***1**–**3***

**Figure 1:**
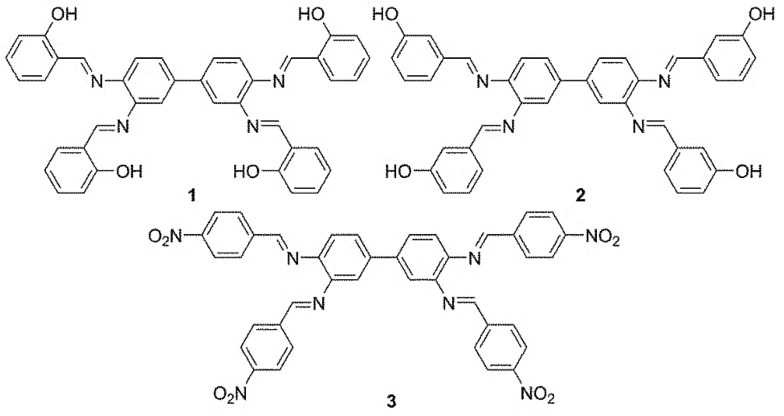
Schiff bases **1**–**3**.

Schiff bases **1**–**3** (Figure 1) were prepared as reported [27] from reaction of biphenyl-3,3′,4,4′-tetraamine and aryl aldehydes, in a ratio of 1:4, in boiling ethanol containing acetic acid as a catalyst. The spectral data of **1**–**3** were in agreement with those reported [27].

### 2.3. Preparation of PS Films

PS (5 g) in chloroform (100 mL) was stirred for 90 min at 25 °C. Schiff bases **1**–**3** (25 mg; 0.5% by weight) were added to a PS solution and the mixture was stirred for 30 min at 25 °C. The homogeneous mixture was cast into clean glass slides (ca. 40 µm thicknesses) and dried for 24 h at 25 °C. The concentration of Schiff bases was chosen as 0.5% by weight to PS based on our previous studies [27,28].

### 2.4. Fourier Transform Infrared Spectroscopy of PS Films

The FT-IR spectra of the PS films were recorded and the carbonyl group index (*I*_s_) was calculated using Equation (1) based on the absorbance of the carbonyl group (*A_s_*) and that for the reference band (*A_r_*) [34].

(1) $$\;I_{s}=A_{s}/A_{r}\;$$

### 2.5. Weight Loss of PS Films

The PS weight loss (%) upon irradiation was calculated using Equation (2) based on the weight of the PS film before (*W*_1_) and after irradiation (*W*_2_) [34].

(2) $$\;Weight\;loss\;\%\;=\;\left[\left(W_{1}-W_{2}\right)/W_{1}\right]\;\times \;100\;$$

### 2.6. Viscometry of PS Films

The average PS molecular weight (${\bar{M}}_{V}^{\alpha }$) was calculated using Equation (3) based on the intrinsic viscosity, [η], and constants *K* and α [35].

(3) $$\;\left[\eta \right]=K{\bar{M}}_{V}^{\alpha }\;$$

## 3. Results and Discussion

### 3.1. IR Spectroscopy of PS

**Figure 2:**
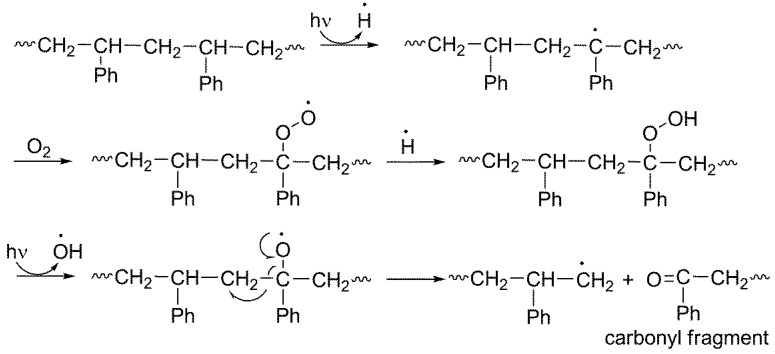
Formation of carbonyl fragments due to PS photo-oxidation.

PS underwent photo-oxidative degradation when exposed to UV irradiation in the presence of oxygen for a long period. Such a process led to a change in the electrical, optical, mechanical and chemical properties of the polymer [6]. In addition, it led to the production of free radicals, cross linking, C–C bonds permanent cleavage and the formation of small fragments containing various functional groups (e.g., OH, C=C and C=O) [36,37]. Figure 2 shows a possible pathway for the formation of carbonyl fragments from the photo-oxidation of PS [38].

**Figure 3:**
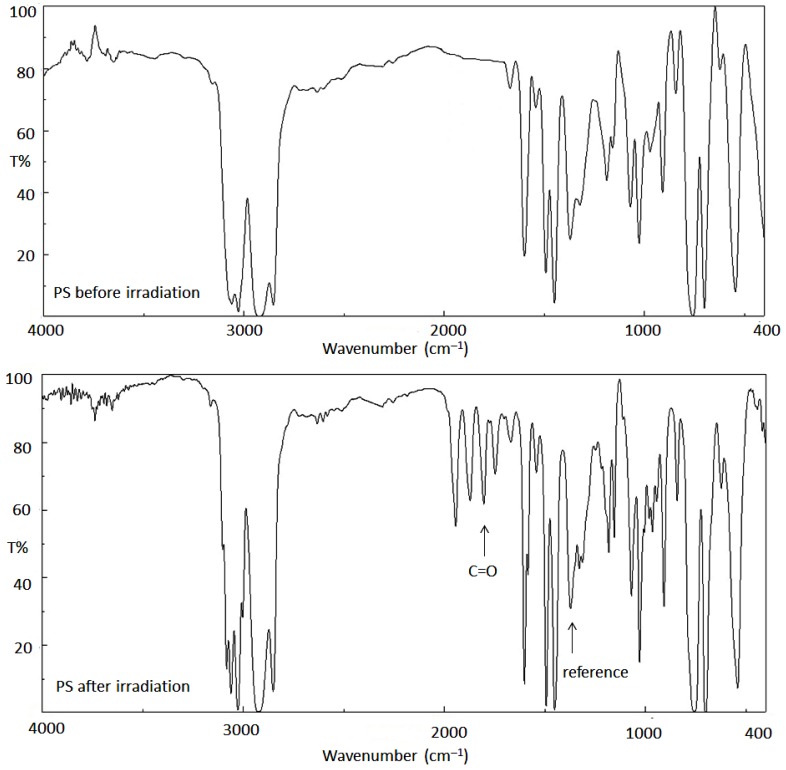
FT-IR spectra of PS film [17].

The intensity of the signal corresponding to the carbonyl group in the IR spectrum of PS was able to give an indication of the rate of photodegradation. Therefore, the FT-IR spectra of the blank PS film and the ones containing Schiff bases **1**–**3** (0.5 wt %) were recorded before and after irradiation (300 h). The FT-IR spectra of the PS film (blank; 40 µm) recorded at an irradiation time of 0 and 300 h are shown in Figure 3 [17].

**Figure 4:**
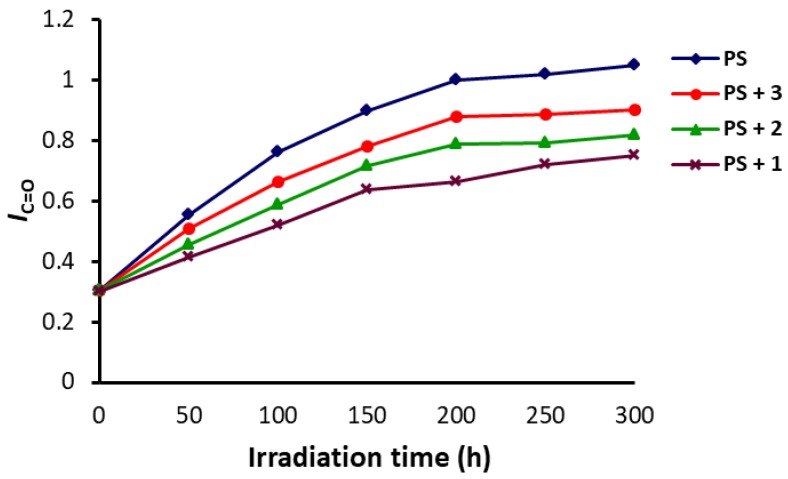
Changes in the *I*_C=O_ of PS upon irradiation.

It was clear that the intensity for the signal corresponding to the absorption of the C=O group (1720 cm^−1^), in the FT-IR for the PS film after irradiation, was much higher than the corresponding one before irradiation. The peak corresponding to the C–C bonds (1328 cm^−1^) was used as a reference peak for comparison [34]. The carbonyl group index (*I*_C=O_) was calculated using Equation (1). Figure 4 shows the changes observed in the *I*_C=O_ when irradiation time ranged from 0 to 300 h. It was clear that PS films containing Schiff bases (0.5 wt %) showed lower carbonyl group indices upon irradiation in comparison to the one for the blank PS film. The changes in *I*_C=O_ were sharp in the first 100 h of irradiation, while the changes were minimal in the last 100 h (200–300 h). The carbonyl group index was 1.05 for the PS film (blank) and 0.75 for the PS/Schiff base **1** blend, after 300 h of irradiation. Such results confirmed the effective use of the Schiff bases and in particular Schiff base **1** to enhance the PS films photostability.

**Figure 5:**
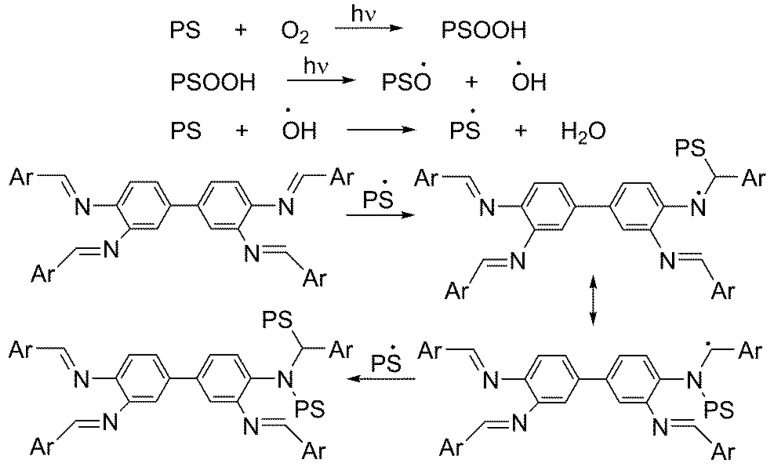
Photostabilization of PS in the presence of Schiff bases **1**–**3**.

Schiff bases **1**–**3** are highly aromatic since they contain four aryl rings and can act as efficient UV absorbers [39]. In addition, additives **1**–**3** can produce stable complexes with the PS radicals in the presence of a chromophore [39]. Figure 5 shows possible pathways for the photostabilization of the PS radicals obtained in the photo-oxidation process in the presence of additives used.

The highest photostabilization for the PS films was seen when Schiff base **1** was used as the additive. Schiff base **1** contained an *ortho*-hydroxy groups at the 2-position of the aryl ring next to the CH=N bonds. Such an arrangement led to better absorption of the energy from the UV light [27]. In addition, it led to dissipation of absorbed energy over time at a harmless rate to the PS chains via a series of processes including internal conversion, intersystem conversion and proton transfer [18,40]. Therefore, Schiff base **1** was considered to be an efficient photostabilizer and had the ability to reduce the photodegradation rate of PS.

### 3.2. Weight Loss of PS

**Figure 6:**
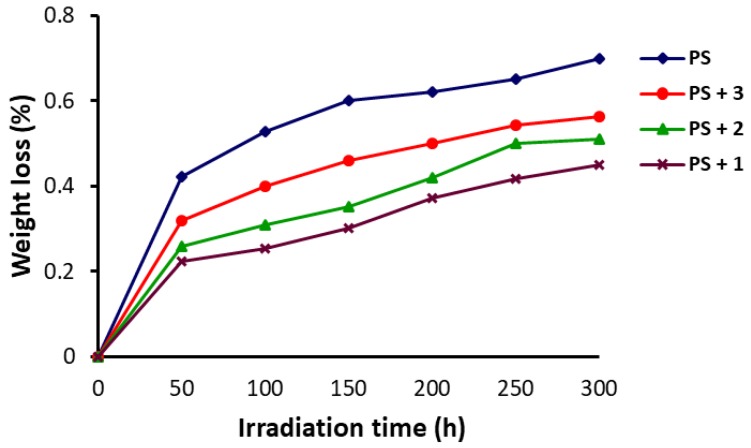
Changes in the weight of PS upon irradiation.

Polystyrene undergoes rapid color change from colorless to yellow and a gradual embrittlement when exposed to UV light for a long term at a high temperature, leading to small fragments and polymer weight loss [41]. The PS films were irradiated with a UV light for up to 300 h and the weight loss was calculated using Equation (2). The changes in the PS weight upon irradiation (300 h) are represented in Figure 6. The weight loss increased sharply in the first 50 h and then gradually up to 300 h. It was clear that the polymer weight loss was higher for the blank PS film in comparison to the blends of PS and Schiff bases **1**–**3**. Schiff base **1** showed the least weight loss compared to the other Schiff bases used.

### 3.3. Molecular Weight of PS

**Figure 7:**
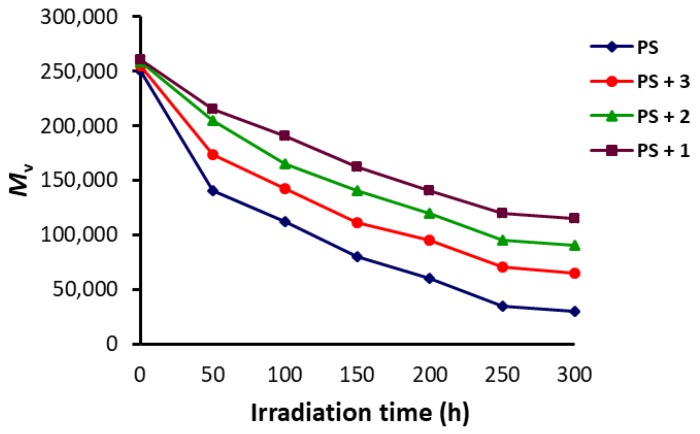
Changes in the ${\bar{M}}_{V}$ of PS upon irradiation.

The viscosity average molecular weight (${\bar{M}}_{V}$) for the PS (in solution) can be calculated using Equation (3), known as the Mark-Houwink equation [42,43]. Such an equation can be used for various ranges of polymers, but is not applicable for low molecular weight ones. The ${\bar{M}}_{V}$ of PS was expected to decrease when irradiation time increased as a result of branching and cross-linking of polymeric chains [44]. The effect of Schiff bases **1**–**3** (0.5 wt %) on the ${\bar{M}}_{V}$ variation for PS was tested. The polymeric materials were irradiated (0–300 h) and the variation in ${\bar{M}}_{V}$ was calculated (Figure 7).

Clearly, the photodegradation of the blank PS was very significant and the reduction in the ${\bar{M}}_{V}$ was very sharp in the first 50 h. The variation in the ${\bar{M}}_{V}$ was very significant for the blank PS and reduced from ca. 250,000 to only 30,000 after 300 h of irradiation. The reduction in the ${\bar{M}}_{V}$ for the PS films containing **1**–**3** was in the range of ca. 65,000–115,000 after 300 h of irradiation. Schiff base **1** was the most effective additive as a photostabilizer for PS since the ${\bar{M}}_{V}$ was reduced from ca. 250,000 (before irradiation) to 115,000 after 300 h of irradiation.

**Figure 8:**
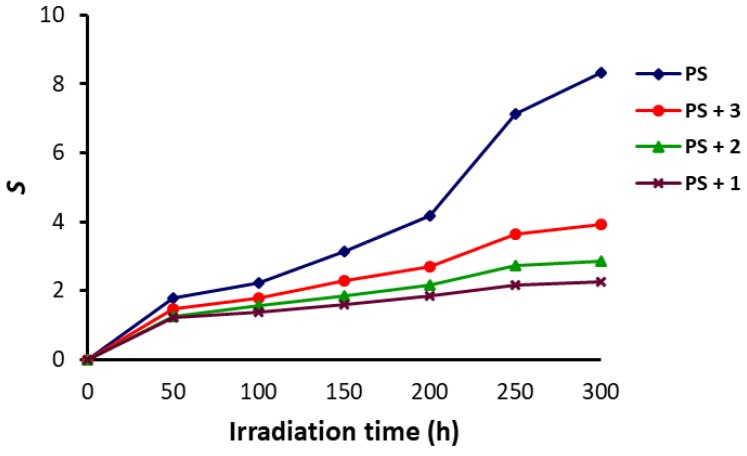
Changes in the *S* of PS upon irradiation.

Impurities within the PS, such as small aromatics, olefins and peroxides, could initiate the formation of radicals that were responsible for the photodegradation and photo-oxidation of the polymeric chains. The most common photodegradation reaction is known as the chain scission [45]. Therefore, the calculation of the chain scission (*S*) for the PS would provide evidence for the degree of its photodegradation. Equation (4) was used to calculate the *S* values based on the ${\bar{M}}_{V}$ at the beginning of irradiation and at a time *t*. The *S* values for the PS films were found to be dependent on the time of irradiation (Figure 8) and increased as irradiation time (0–300 h) increased. The *S* values were much lower for the PS/Schiff bases in comparison to the blank film. For example, the *S* value was 8.3 for the blank PS film after 300 h of irradiation compared to only 2.3 for the PS film containing Schiff base **1** for the same length of time. Clearly, the use of Schiff bases as additives reduced the photodegradation of PS to a significant level.

(4) $$\;S={\bar{M}}_{V,O\;}/{\bar{M}}_{V,t}\;-1\;$$

**Figure 9:**
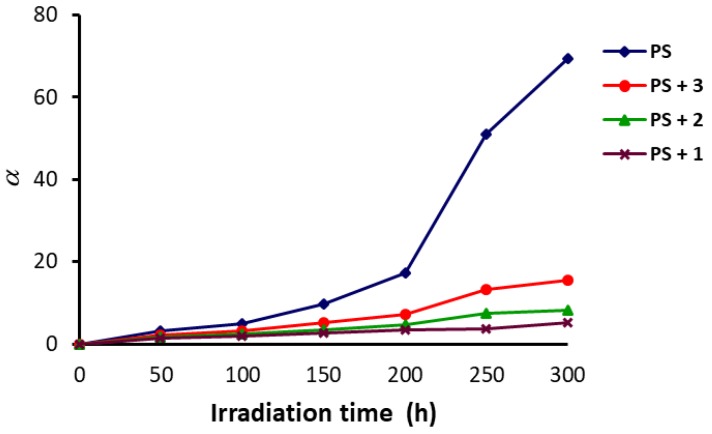
Changes in the *α* of PS upon irradiation.

The photodegradation process of polymers leads to high deterioration (*α*) as a result of weak bonds breaking at the beginning of the process. Equation (5) was used to calculate the *α* values which were directly proportional to the PS molecular weight (*m*) and *S* and inversely proportional to ${\bar{M}}_{V}$. Therefore, it was expected that *α* would increase upon increasing irradiation time for the PS films. Figure 9 shows that *α* increased dramatically upon increasing irradiation time for the blank film in comparison to the blends of the polymer and additives. For example, *α* was 96 for the blank PS film and only 5 for the PS film containing Schiff base **1**, after 300 h of irradiation.

(5) $$\;\alpha =m.S/{\bar{M}}_{V}\;$$

### 3.4. Microscopic Surface Morphology of PS

**Figure 10:**
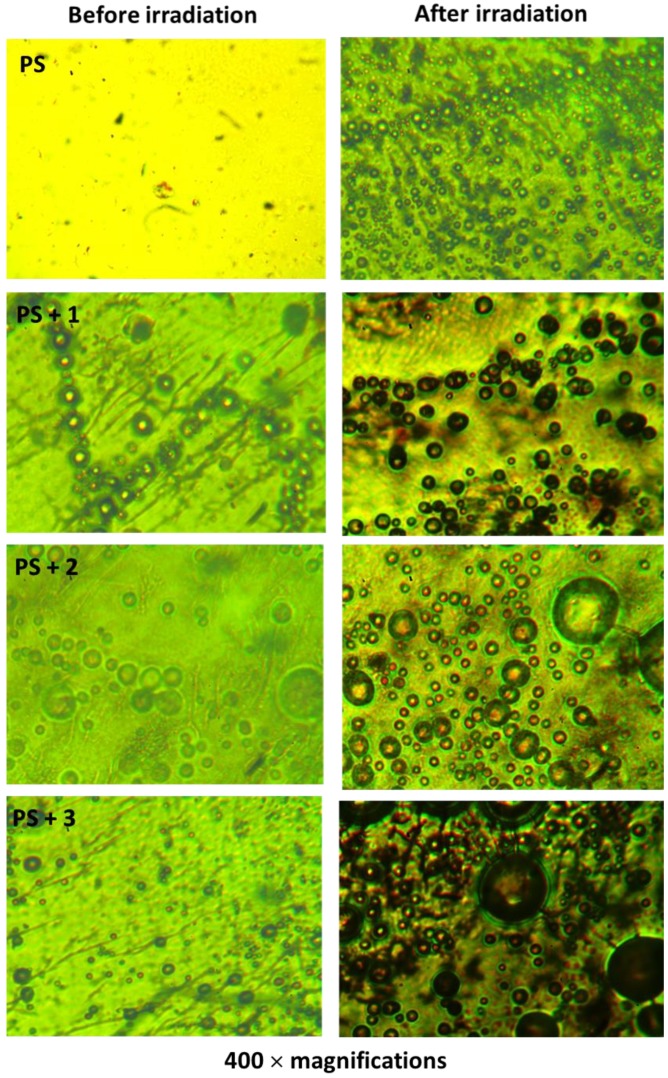
Microscopic images of PS and PS/Schiff bases **1**–**3** blends before and after irradiation.

UV absorbers are capable of reducing the photodegradation and photo-oxidation processes of PS through direct absorption of harmful radiation [6]. The surface morphology (400× magnifications) of the PS films was examined by microscope before and after irradiation (Figure 10). The microscopic images of the non-irradiated films showed a smooth surface with no or a limited number of white spots and grooves. For the irradiated PS films, the microscopic images showed the presence of rough surface and various surface crazes. However, the number of white spots and groves were low in the films containing additives compared to the PS film (blank) which proved the effective use of Schiff bases **1**–**3** as photostabilizers for the PS films.

### 3.5. Scanning Electron Microscopy (SEM) of PS

**Figure 11:**
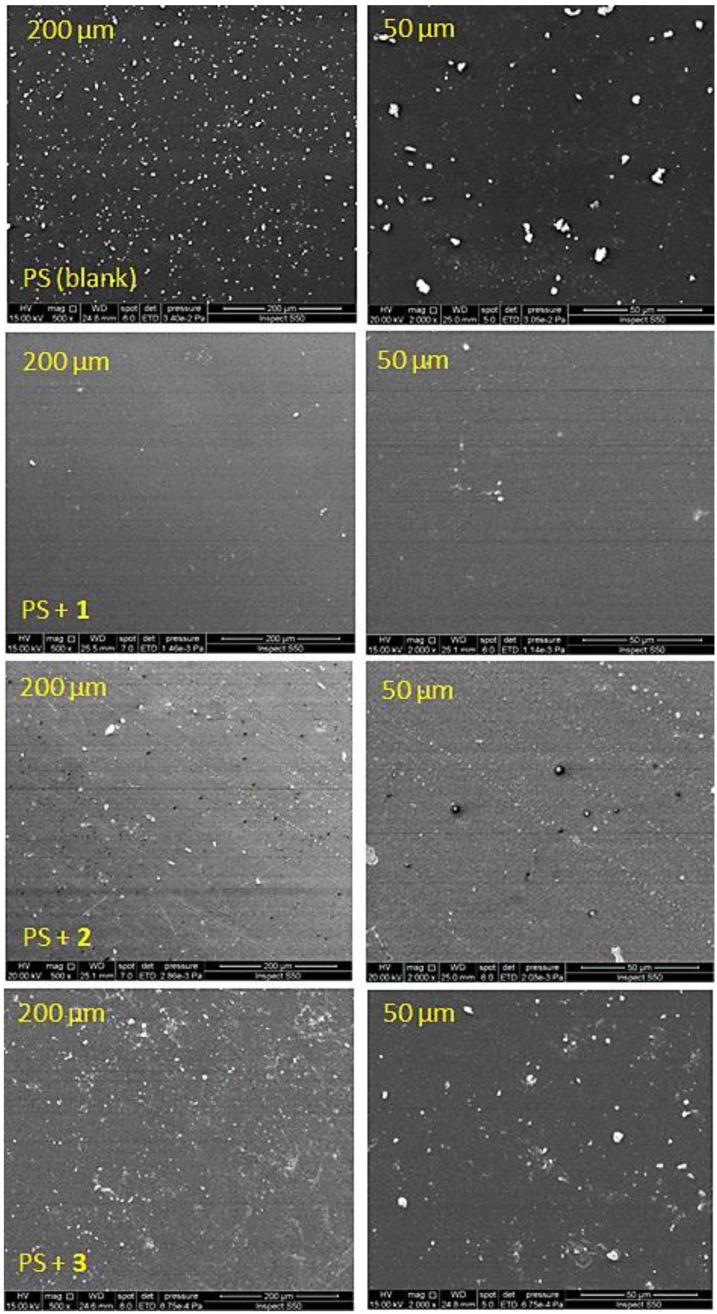
SEM images of PS and PS/Schiff bases **1**–**3** blends before irradiation.

**Figure 12:**
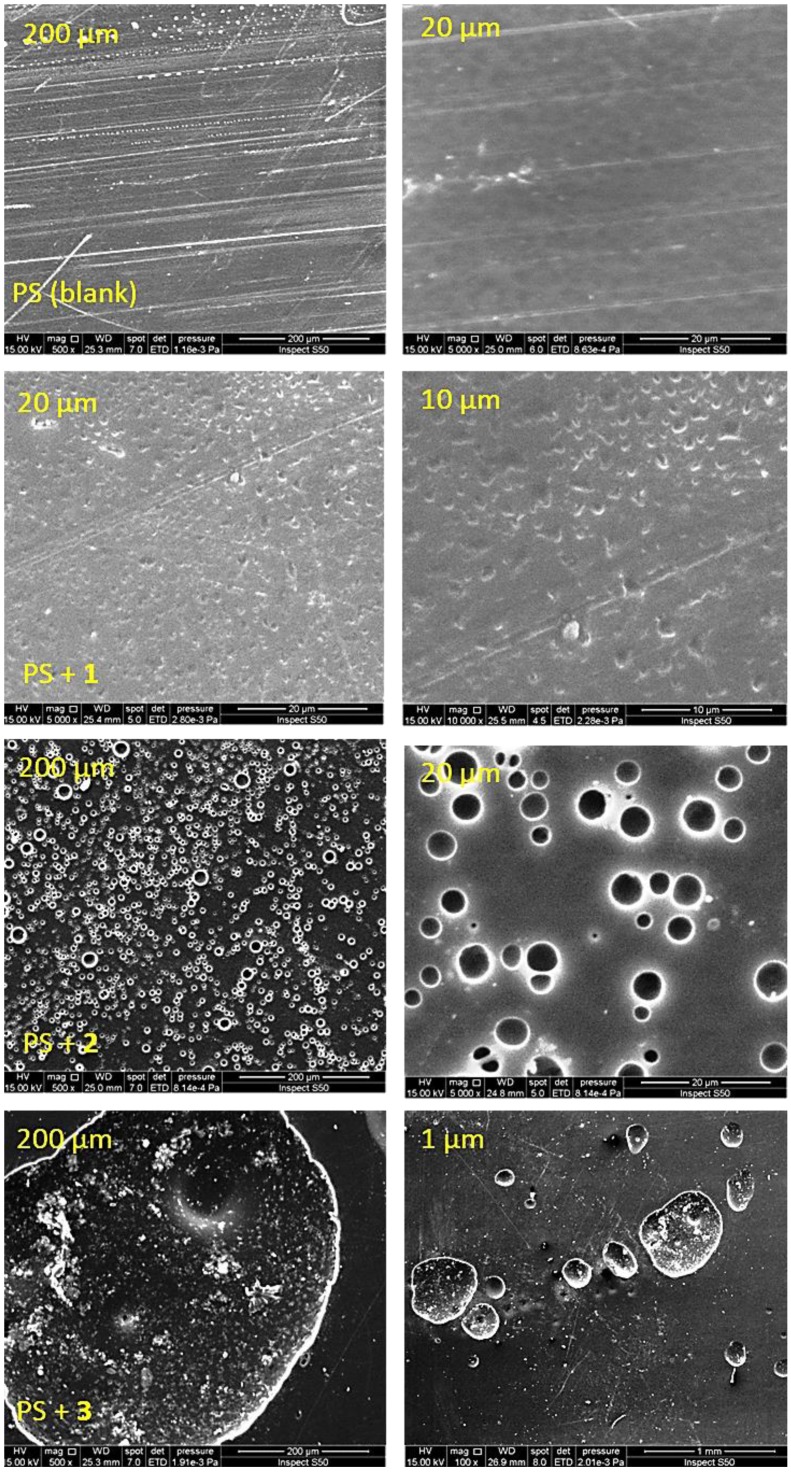
SEM images of PS and PS/Schiff bases **1**–**3** blends after irradiation.

SEM provides useful information about the polymers’ surface morphology that in turn reflects their internal structures [46]. Clear magnified images for the PS surface can be taken using electron beams. The surface of the PS films was investigated using the SEM (15 KV) and the images recorded at different magnification powers (Figure 11 and Figure 12). The SEM images for the non-irradiated PS films showed a smooth and clean surface with grain boundaries and high particles homogeneity (Figure 11). After 300 h of UV irradiation, the incorporation of the Schiff bases within the PS led to a general change in the particle size and their random distribution on the surface. The SEM images for the PS films showed an almost rough surface after irradiation. Clearly, there was a drastic change in the PS surface morphology upon irradiation. The images for PS/Schiff bases (0.5 wt %) blends suggested that irradiation caused only minor damage to the compact texture (Figure 12). For the films containing additives, there was a clear sign for the high resistance to irradiation that reflects the high chemical stability of the blends. In addition, the SEM images for the PS/Schiff bases blends showed small balls that varied in size (ca. 3.4–4.3 μm diameter) and shape (sphere and embedded ellipsoid). The balls-like phenomena could be due to the high light absorption and multi-porous structure of the additives [47].

### 3.6. Atomic Force Microscopy (AFM) of PS

**Figure 13:**
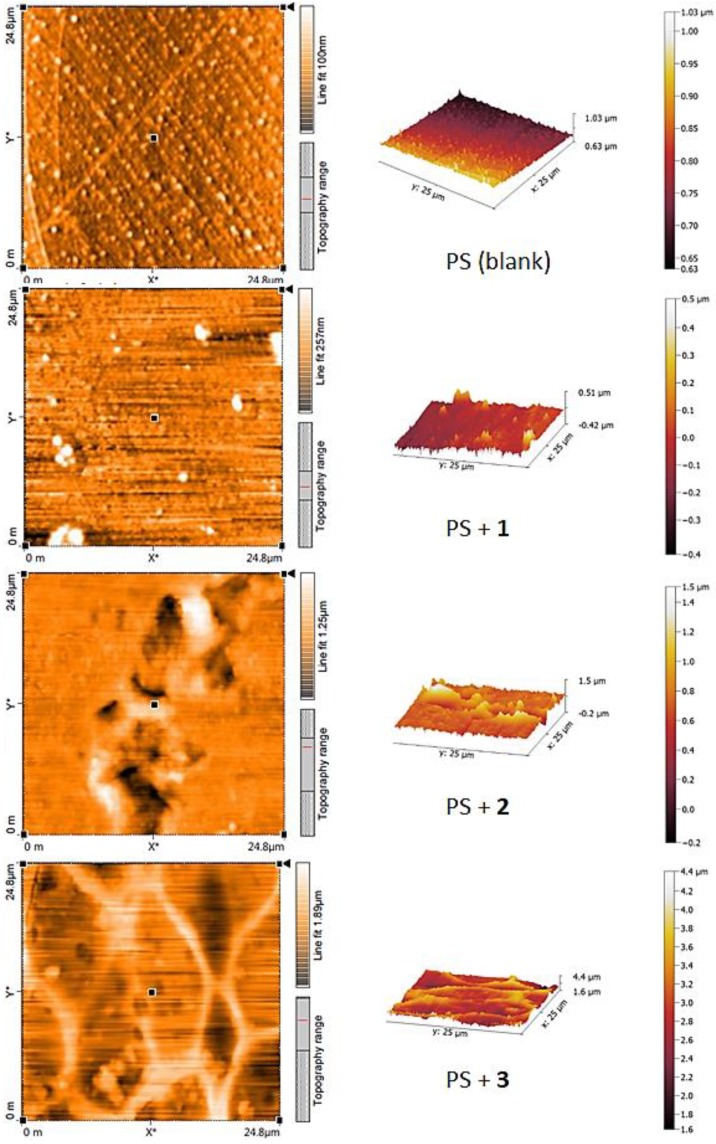
2D and 3D AFM images of PS and PS/Schiff bases **1**–**3** blends before irradiation.

**Figure 14:**
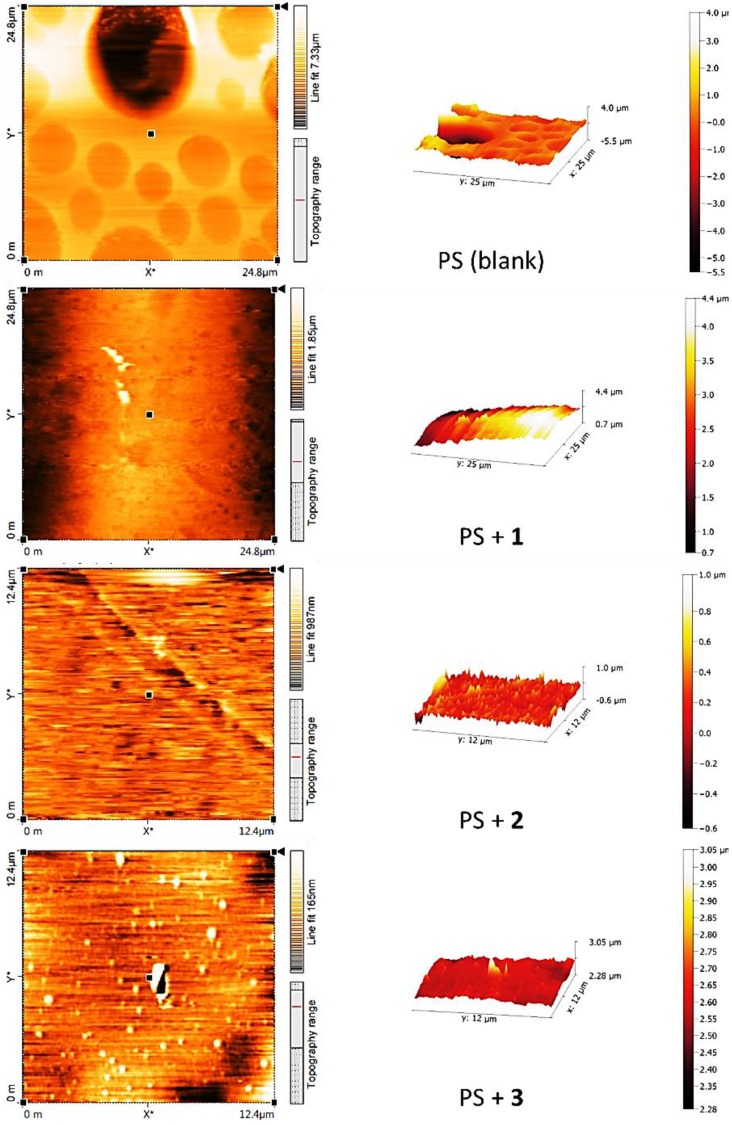
2D and 3D AFM images of PS and PS/Schiff bases **1**–**3** blends after irradiation.

AFM is a high resolution scanning microscope that can be efficiently used to investigate the surface morphology and particles of materials [48,49,50]. Therefore, the AFM surface analysis of the PS films (surface area = 4.0 × 4.0 μm^2^) before and after irradiation were recorded as shown in Figure 13 and Figure 14, respectively.

The two- and three-dimensional AFM images indicated that the PS films showed a smooth surface (Figure 13). After irradiation, the blank PS film has a rough surface which is an indication for a significant degree of photodegradation (Figure 14). The AFM images for the PS films containing additives showed a much smoother and more or less uniform surface compared to the surface of the blank PS film. Schiff base **1** was very effective in inhibiting photodegradation of PS compared to the other Schiff bases **2** and **3**. Clearly, the surface of the PS film containing **1** was very smooth and uniform (Figure 14). The AFM images for the PS containing Schiff bases **2** and **3** showed an area of roughness and featureless. The surface roughness was 346.3 nm for the blank PS film compared to 41.6, 77.0 and 80.2 nm for the PS/**1**, PS/**2** and PS/**3** films, respectively. In addition, the AFM images for the PS/additive blends indicated formation of particles that have a sub-micron size, which is in agreement with the results obtained from the SEM study (Figure 12).

## 4. Conclusions

A novel ball-like polystyrene/Schiff base microspheres at a low concentration were facilely synthesized using the casting method. Irradiation of polystyrene films containing Schiff base additives for long periods leads to the formation of ball-like microspheres. The SEM images of the polystyrene surface showed clearly that the diameter of the balls was in the range 3.4–4.3 µm. The photodegradation process of polystyrene was reduced significantly when Schiff bases were mixed within the films. The Schiff bases used acted as polystyrene photostabilizers and could have the potential to be used on a commercial scale.
